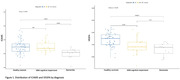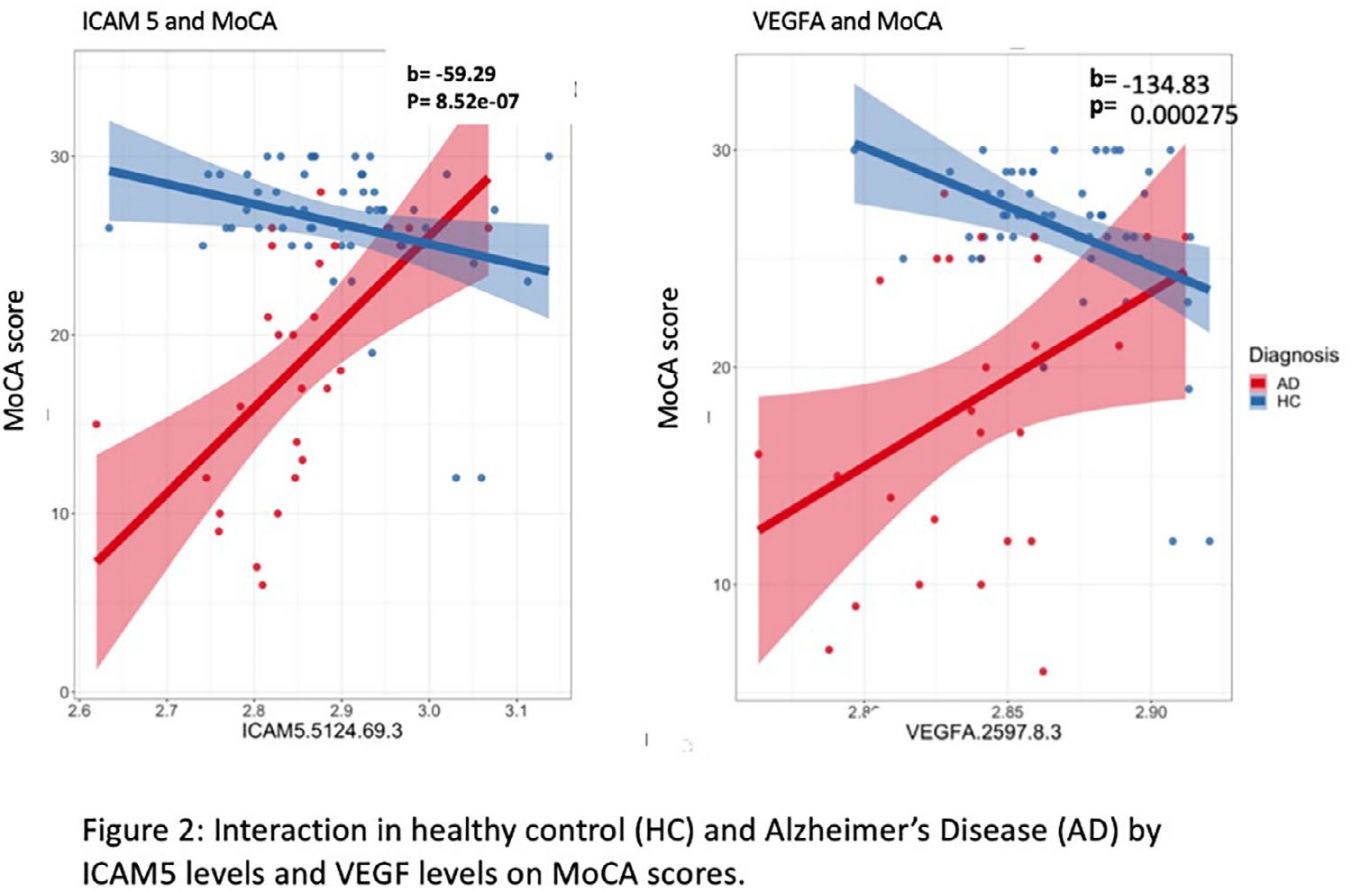# VEGF and ICAM5 Levels in Alzheimer’s Disease and Cognitively Healthy Aging

**DOI:** 10.1002/alz.088503

**Published:** 2025-01-03

**Authors:** Claire Delpirou Nouh, Olamide Abiose, Tony Wyss‐Coray, Victor W Henderson, Sharon J Sha, Maya Yutsis, Elizabeth Mormino, Kyan Younes

**Affiliations:** ^1^ Department of Neurology and Neurological Sciences, Stanford University, Stanford, CA USA; ^2^ Wu Tsai Neurosciences Institute, Stanford University, Stanford, CA USA; ^3^ Department of Neurology and Neurological Sciences, Stanford University School of Medicine, Stanford, CA USA

## Abstract

**Background:**

Vascular dysfunction, blood‐brain barrier (BBB) dysregulation, and neuroinflammation are thought to participate in Alzheimer`s disease (AD) pathogenesis, though the mechanism is poorly understood. Among pathways of interest, AD pathology appears to affect vascular endothelial growth factor‐A (VEGFA) signaling in a bidirectional manner. Higher VEGF levels are thought to have a protective role and slow cognitive decline. Neuronal intracellular adhesion molecules (ICAMs) may also be a marker of BBB permeability. ICAMs participate in the immune‐nervous system interactions, synaptic plasticity, and cognition.

**Method:**

We used the SomaLogic platform for cerebrospinal fluid (CSF) proteome profiling to investigate the levels of ICAM 1, 2, 3, 5 and VEGFA as markers of vascular dysfunction and neuroinflammation in 83 participants from the Stanford Alzheimer Disease Research Center cohort (54 cognitively unimpaired healthy controls (HC) and 29 with mild cognitive impairment or dementia due to AD). All samples were blinded for analysis. Montreal Cognitive Assessment (MoCA) was used as a measure of global cognition. ANOVA compared the levels of biomarkers in diagnostic groups. Linear regression and interaction terms of group (AD vs HC) by biomarker levels were used to investigate the effect on global cognition.

**Result:**

ICAM5 and VEGFA were significantly associated with cognition in our cohort. Both ICAM5 and VEGFA levels were lower in AD compared to HC (Figure 1). Interaction terms of diagnosis by ICAM5 were significant on MoCA (b = ‐59.29, p‐value < .0001), and of diagnosis by VEGFA were also significant on MoCA (b = ‐134.8, p:0.00027), indicating a divergent role for these biomarkers in AD vs. HC (Figure 2). Other ICAMs were not significantly associated with cognition in our cohort.

**Conclusion:**

VEGFA and ICAM5 may play an important and dynamic role in AD. More research is needed to understand the interplay between ICAM5 and VEGF and their relationship with AD.

**References**: Tao QQ et al. Aging Dis. 2022; Tubi MA et al. Neurobiol Aging. 2021; Petrelis AM et al. Aging (Albany NY). 2022; Yuan L et al. Aging Dis. 2017; Hino H et al. Brain Res. 1997; Yang H. Comp Funct Genomics. 2012; Birkner K et al. Front Neurol. 2019